# Revised estimates of influenza-associated excess mortality, United States, 1995 through 2005

**DOI:** 10.1186/1742-7622-5-26

**Published:** 2008-12-30

**Authors:** Ivo M Foppa, Md Monir Hossain

**Affiliations:** 1 Department of Epidemiology, Tulane University School of Public Health and Tropical Medicine, New Orleans, LA 70112, USA; 2 Center for Clinical and Translational Sciences, University of Texas Health Science Center at Houston, Houston, TX 77030, USA

## Abstract

**Background:**

Excess mortality due to seasonal influenza is thought to be substantial. However, influenza may often not be recognized as cause of death. Imputation methods are therefore required to assess the public health impact of influenza. The purpose of this study was to obtain estimates of monthly excess mortality due to influenza that are based on an epidemiologically meaningful model.

**Methods and Results:**

U.S. monthly all-cause mortality, 1995 through 2005, was hierarchically modeled as Poisson variable with a mean that linearly depends both on seasonal covariates and on influenza-certified mortality. It also allowed for overdispersion to account for extra variation that is not captured by the Poisson error. The coefficient associated with influenza-certified mortality was interpreted as ratio of total influenza mortality to influenza-certified mortality. Separate models were fitted for four age categories (<18, 18–49, 50–64, 65+). Bayesian parameter estimation was performed using Markov Chain Monte Carlo methods. For the eleven year study period, a total of 260,814 (95% CI: 201,011–290,556) deaths was attributed to influenza, corresponding to an annual average of 23,710, or 0.91% of all deaths.

**Conclusion:**

Annual estimates for influenza mortality were highly variable from year to year, but they were systematically lower than previously published estimates. The excellent fit of our model with the data suggest validity of our estimates.

## Background

**Figure 1:**
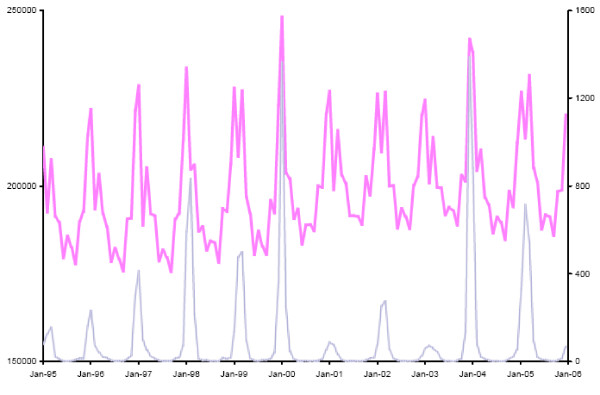
**All-Cause and Influenza Mortality, U.S., 1995–2005**. Monthly all-cause (primary y-axis, pink) and influenza mortality (secondary y-axis, light blue), United States, 1995 to 2005. Monthly data points are connected by straight lines for better visualization.

Influenza viruses, due to their genotypic plasticity [1], cause yearly epidemics that generally coincide with peaks in all-cause mortality (Figure 1). Incidence of these infections is difficult to quantify because of their clinical similarity with other upper respiratory infections and because laboratory confirmation is rarely done. Mortality due to seasonal influenza, which may result from exacerbation of underlying pulmonary, cardiac or other systemic conditions is, nevertheless, thought to be substantial [2-17]. Recent U.S. estimates of average annual excess mortality due to seasonal influenza exceed 30,000 [11,14].

To estimate excess mortality due to influenza, two fundamental approaches have previously been used. The most popular one is based on Serfling's seasonal regression method [18] and has resulted in numerous estimates of excess mortality due influenza [3-8,12,14,15,19]. This periodical regression approach is based on parametric estimation of a sinusoidal "baseline" function that represents mortality in absence of influenza. The difference between the baseline function and the observed numbers of deaths is then interpreted as the number of excess deaths due to influenza. Typically, the baseline mortality function is fitted to weekly or monthly mortality rates or numbers during non-influenza months, using two or more Fourier terms [18]. This approach is intuitively appealing as it captures the strong seasonal periodicity of mortality. However, the particular choice of a parametric baseline function lacks epidemiological justification: Why should the baseline function be sinusoidal rather than of any other periodic form? Depending on the shape of the "true" baseline function, under- or overestimation of excess mortality due to influenza might result. If, for example, the true baseline function is "higher" (i.e. the definite integral of the true function is larger) than the assumed sinusoidal function, then overestimation would result and *vice versa*. Another, potentially more important shortcoming of the periodical regression approach lies in the fact that seasonally correlated causes of mortality, including influenza, are not controlled for, which might lead to confounded estimates of excess mortality.

To avoid this difficulty, one could gauge all-cause mortality with some independent measure of influenza transmission (or mortality). Following this rationale, Thompson et al. [11] estimated excess mortality due to both influenza and respiratory syncytial virus (RSV). They used a generalized linear model (GLM) with a Poisson distribution and a logarithmic link function to model the weekly number of deaths. They also used two Fourier terms in their model, but, in addition, used indicators of influenza and RSV transmission. These indicator variables were defined by the proportions of specimens testing positive for influenza A(H1N1), influenza A(H3N2), influenza B and RSV. Several potential shortcomings of this methods are, however, apparent. First, this model also makes *a priori *assumptions about the baseline mortality function–in this case an exponentiated sinusoidal function. Although this might conceivably be true, there is little empirical evidence to support this assumption. Second, the multiplicative form of the model implies that excess mortality, given a certain amount of influenza activity, depends on the current level of all-cause mortality. Again, this does not appear to be a well-founded assumption. Finally, the proportion of test positive specimens is likely to be a poor measure of excess mortality. While a high proportion of test positive specimens is compatible with high levels of influenza transmission (and excess mortality), this is not necessarily true. The model, however, implies that five hundred influenza positives, obtained from a thousand tests, are associated with less excess mortality than two influenza positives, obtained from three tests. This appears to be an unrealistic assumption. The seasonally changing frequency of influenza testing [20] is, at least partly, due to the seasonally changing incidence of other agents causing influenza-like illness (ILL).

Alternatively, one could postulate that mortality directly attributed to influenza (influenza-certified mortality) represents a certain proportion of all mortality attributable to influenza. This assumption implies that the coefficient associated with influenza-certified mortality represents the ratio of total influenza mortality to influenza certified mortality [17,21]. Here we use a method for the estimation of influenza excess mortality which is similar to the one recently presented by Schanzer and colleagues [17]: we adopt the proportionality assumption and avoid specific parametric assumptions about the baseline function. In addition, and deviating from the Schanzer model, we allow for random variability of influenza-certified mortality by adopting a hierarchical modeling approach. We present the resulting estimates of U.S. excess mortality due to influenza for the years 1995 to 2005. We compare these to estimates obtained from a Thompson-like model [11], as well as to previously published estimates of influenza-associated excess mortality.

## Methods

### Data

We used Multiple Cause-of-Death Data for the years 1995 to 2005 (Multiple Cause-of-Death Microdata, 1995–2005, National Center for Health Statistics, Hyattsville, Maryland). This dataset is in the public domain and can be electronically downloaded from the web site of the National Bureau of Economic Research . We defined deaths as influenza-certified if influenza was given as underlying cause of death. The corresponding diagnostic code for ICD-9 (1995 to 1998) was 487 and and for ICD-10 (1999 onwards) the code range was J10–J12. Influenza years were defined as lasting from July 1 of one year to June 30 of the following year. We defined four age categories: < 18, 18–49, 50–64 and 65+. Observations with missing age (N = 4,490) were not included in this analysis.

### Statistical model

The epidemiological model on which our analyses are based implies that monthly all-cause mortality is the sum of "baseline mortality", i.e. mortality that is independent of influenza, and mortality that is a direct or indirect result of influenza. Based on the pronounced seasonal periodicity of all-cause mortality we assume that baseline mortality is a function of calender month. In addition, we allow for a linear and/or non-linear trend in all-cause mortality that takes into account demographic or other population level changes resulting in linear/non-linear changes in baseline mortality over time. Finally, we accommodate extra variability of baseline mortality that is not accounted for by calender month and trend. This epidemiological model corresponds to the following statistical model, hence referred to as "current model":

(1) *Y*_*i*_|*θ*_*i *_~ Poisson(*θ*_*i*_)

(2) *θ*_*i *_~ Normal(*μ*_*i*_, *τ*)

(3) $$\mu_{i}=\lambda_{m_{i}}+t_{i}\beta_{1}+t_{i}^{2}\beta_{2}+t_{i}^{3}\beta_{3}+\gamma_{i}\phi$$

(4) *X*_*i*_|*γ*_*i *_~ Poisson(*γ*_*i*_),

where *Y*_*i *_is the observed all-cause mortality count during index months *i *= 1, ..., 132. The variable *Y*_*i *_which represents a number and not a rate, is assumed to follow a Poisson distribution with a mean parameter *θ*_*i*_. The Poisson mean parameter *θ*_*i *_has an identity link and is distributed as Normal with mean *μ*_*i *_and variance *τ*. This parametrization for the Poisson mean *θ*_*i *_allows for overdispersion. In the implementation, *θ*_*i *_is restricted to positive values to ensure the positivity of the generated samples. The model for *μ*_*i *_has two parts. The first part concerns mortality due to non-influenza related causes (baseline mortality) which includes a random intercept $\lambda_{m_{i}}$ for calendar month *m*_*i*_(*m*_*i *_= 1, ..., 12) that models the seasonal background mortality, and also includes linear, quadratic and cubic effects for temporal changes due to health, demographic or socioeconomic factors. The variable *t*_*i *_(*t*_*i *_= 0, ..., 10) indicates the calendar year; *t*_*i *_= 0 corresponds to the year 1995. The regression coefficients *β*_1_, *β*_2 _and *β*_3 _measure these changes. The second part of the model for *μ*_*i *_concerns mortality due to influenza. The symbol *γ*_*i *_is the Poisson parameter from the second level of hierarchy for the observed influenza-certified mortality, *X*_*i*_. The parameter *ϕ *measures the effect of influenza-certified mortality on all cause mortality assuming that all other effects are fixed. This is the parameter of interest. It can also be interpreted as the ratio of total influenza mortality to influenza-certified mortality. Thus, the total excess influenza mortality for index month *i*, $X_{i}^{*}$, is given by

(5) $$X_{i}^{*}=\gamma_{i}\phi .$$

To estimate excess mortality due to influenza, ${\hat{X}}_{i}^{*}$ is calculated using expression 5, with posterior estimates of *γ*_*i *_and *ϕ*. As total influenza mortality cannot be lower than influenza-certified mortality, the minimum value for the range in the prior distribution for *ϕ *was set to one (see additional file 1).

To assess the performance of the current model, we also analyzed the data with a modified form of the model proposed by Thompson et al. [11]. The modified model has the following form:

(6) *Y*_*i*_|*μ*_*i *_~ Poisson(*μ*_*i*_)

(7) $$\ln(\mu_{i})=\beta_{0}+t_{i}\beta_{1}+t_{i}^{2}\beta_{2}+\sin(m\frac{2\pi }{12})\alpha_{1}+\cos(m\frac{2\pi }{12})\alpha_{2}+x_{i}\lambda ,$$

where *β*_0 _is an intercept, *β*_1 _and *β*_2 _are defined as above, *α*_1 _and *α*_2 _represent the parameters associated with the Fourier terms and *λ *is the natural logarithm of the rate ratio associated with influenza-certified mortality. In contrast to Thompson et al. we used monthly, rather than weekly data and used observed influenza-certified mortality, rather than proportion of positive influenza tests, as indicator for total influenza mortality. For this Thompson-like (TL) model, because of its multiplicative nature, total excess mortality due to influenza, $X_{i}^{*}$, given by the expression

(8) $$X_{i}^{*}=\mu_{i}-{\bar{\mu }}_{i},$$

where

$$\ln({\bar{\mu }}_{i})=\beta_{0}+t_{i}\beta_{1}+t_{i}^{2}\beta_{2}+\sin(m\frac{2\pi }{12})\alpha_{1}+\cos(m\frac{2\pi }{12})\alpha_{2}.$$

To calculate estimated excess mortality due to influenza, all parameters in 8 are replaced by their posterior estimates

### Statistical analysis

The parameters for this hierarchical model were estimated using a Markov chain Monte Carlo (MCMC) algorithm implemented in WinBUGS, version 1.4.1 (Imperial College and Medical Research Council, UK) [22]. Uninformative prior distributions were used (additional file 1). To ensure positivity of all *θ*_*i*_, the normal priors of this parameter were truncated at non-positive values (additional file 1). The empirical posterior distributions of the parameters were obtained from MCMC samples of 30,000, resulting from three chains with 200,000 burn-in iterations and 10,000 samples each. Posterior means and 95% credible intervals (CIs) were calculated for all parameters of interest after ensuring convergence of all model parameters.

The parameters of the TL model could easily be estimated using a GLM procedure in any standard statistical software package. However, to allow for direct comparison of the model fit we used the same estimation procedure as for the current model. The fit of the two age-specific models was compared using the deviance information criterion (DIC) [23]. DIC penalizes the model goodness-of-fit for additional complexity. The complexity is measured by the effective number of parameters.

In order to quantify the variance explained by the fitted models, we used a Bayesian version of the classical R-squared [24]. i.e.

(9) $$BRSQ=1-\frac{E\left(V_{i=1}^{N}(e_{i})\right)}{V_{i=1}^{N}(Y_{i})},$$

where *E*(·) and *V*(·) are the operators for the posterior mean and empirical variance, respectively and *e*_*i *_= *Y*_*i *_- *μ*_*i*_. The empirical variance of *e *is computed for each iteration.

**Figure 2:**
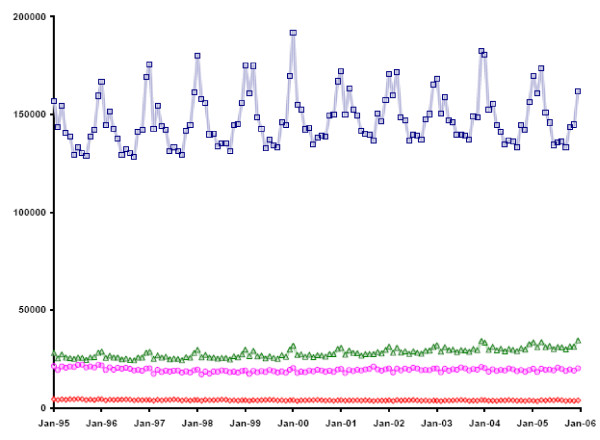
**All-Cause Mortality, Age Categories <18, 18–49, 50–64 and 65+, U.S., 1995–2005**. Monthly all-cause mortality, United States, 1995 to 2005, in the four age categories <18 (red), 18–49 (pink), 50–64 (green) and 65+ (blue).

Separate analyses were performed for the four age categories, because of substantial differences in seasonality of all-cause mortality: While seasonal periodicity is quite obvious in the oldest category, it distinctly decreases with age and becomes inapparent in the youngest category (Figure 2).

## Results

**Figure 3:**
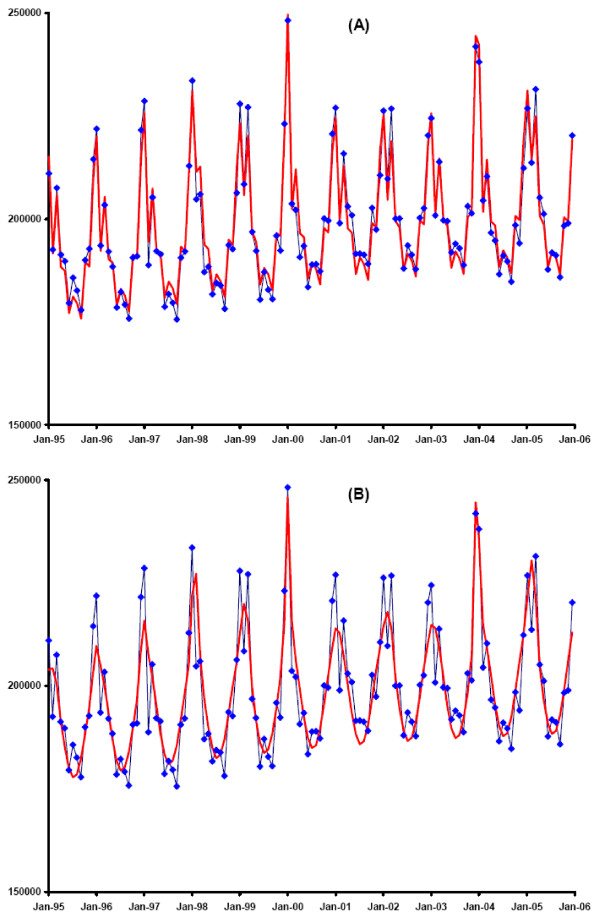
**a & b – Observed and Predicted All-Cause Mortality According to the Current and TL Model, U.S., 1995–2005**. Predicted (red) and observed (blue) monthly all-cause mortality in ages 50 and above, United States, 1995 to 2005. The predicted values are based on the combined means from 30,000 MCMC simulations for each age category, using the current model (expressions 1 through 4) (a) and the TL model (expressions 6 and 7) (b).

A total of 26,262,147 deaths over the period of eleven years were included in this analysis. Of these deaths, 12,922 (0.05%) were influenza-certified. The current model (1–4) fit the data very well (Figure 3a) and explained most of the variance of all-cause mortality (BRSQ for age categories < 18, 18–49, 50–64 and 65+: 0.90, 0.93, 0.97 and 0.96, respectively).

The estimated ratio of influenza-certified deaths to all deaths attributable to influenza was less than three and a half in those younger than 18, over twenty for 50+ and intermediate in the age category 18–49 (Table 1). Overall, we estimated that 260,814 (95% CI: 201,011–290,556) deaths were attributable to influenza over the whole study period. This corresponds to an annual average of 23,710 excess deaths. Most of these deaths (90.93%; 95% CI: 87.13%–93.67%) occurred in people 65 or older. Far less represented were those between 50 and 64 at time of death (6.61%; 95% CI: 4.56%–8.90%). The two lowest age categories contributed together less than three percent (< 18: 0.83%; 95% CI: 0.40%, 1.31% and 18–49: 1.45%; 95% CI: 0.21%–4.85%) to estimated influenza mortality.

**Table 1:** Age category-specific estimates for the detection ratio *ϕ*.

Age Category	Posterior Mean (95% CI)
< 18	3.47 (1.61–5.40)
18–49	9.60 (1.152–26.95)
50–64	22.96(15.66–30.67)
65+	21.16 (18.06–24.32)

Table 2 shows the estimated number of deaths due to influenza for full influenza years, according to the current model. The numbers varied widely from year to year. By far the largest number of seasonal deaths due to influenza was observed for 2003/04, with close to 47,000 deaths. The lowest numbers, which only little exceeded one tenth of that, were seen for the years 2000/01 and 2002/03.

**Table 2:** Estimated Numbers of Deaths Attributable to Influenza, United States, 1995–2005, according the the current model.

influenza Year	Posterior Mean (95% CI)
1995/96	12,067 (8,594–13,898)
1996/97	19,373 (14,750–21,895)
1997/98	36,778 (29,368–41,555)
1998/99	26,666 (20,813–30,381)
1999/00	43,339 (33,886–46,708)
2000/01	5,479 (3,540–6,434)
2001/02	14,995 (11,256–17,405)
2002/03	5,371 (3,523–6,625)
2003/04	49,925 (39,181–52,919)
2004/05	36,726 (28,914–40,881)

**Figure 4:**
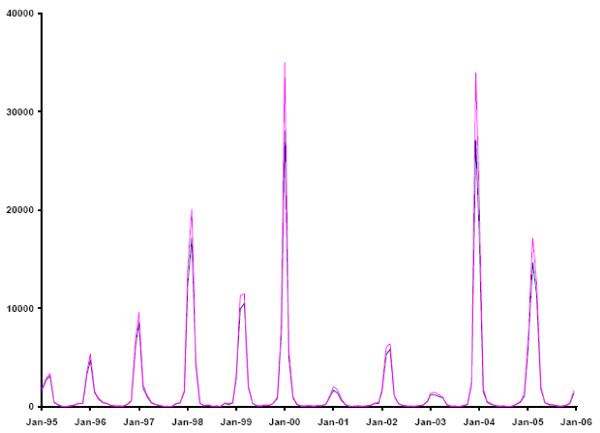
**Predicted Influenza Mortality according to the Current and the TL Model, U.S., 1995–2005**. Predicted total influenza mortality, United States, 1995 to 2005 according to the current (blue) and the TL model (red). The predicted values are based on the combined means from 30,000 MCMC simulations for each age category.

The TL model resulted in 302,665 (95% CI: 294,192–311,200) estimated excess deaths due to influenza, which is 16% higher than our overall estimate (additional file 1). The fit of this model, according to the DIC, was slightly better than the current model for the youngest age category (Table 3), that exhibited little seasonal variation in all-cause mortality. For all other age categories, particularly the oldest one, the fit of the TL model, compared to the current model, was distinctly inferior (Table 3). The fit of the age group-specific TL models combined was worse than the corresponding fit of the current model (Figures 3a and 3b). Accordingly, the variance explained by the TL model was also lower than for our model (BRSQ for age categories < 18, 18–49, 50–64 and 65+: 0.61, 0.31, 0.84 and 0.83, respectively). Yet, the pattern of predicted total influenza deaths was virtually identical to the one predicted by the current model (Figure 4).

**Table 3:** Comparison of the age-specific fit (DIC) of the current with the TL model.

Age Category	Current Model	TL Model
< 18	2,279.30	2,021.39
18–49	2,235.22	6,123.61
50–64	2,298.53	5,656.66
65+	2,912.66	27,298.70

## Discussion

Our estimates of excess mortality due to influenza are substantial, especially for the influenza years 1997/98, 1999/2000 and 2003/04, during which influenza A(H3N2) predominated. The lowest estimates were obtained for the years 2000/01 and 2002/03, when influenza A(H1N1) and B viruses predominated. Nevertheless, our estimates are markedly lower than previous estimates. For the year 1995/96, for example, we attributed 12,067 excess deaths to influenza. For the same period, Simonsen et al. [14] estimated that 25,071 deaths were attributable to influenza in ages 65+ alone. Thompson et al. [11] estimated the number of excess deaths during that influenza year at 36,280–more than three times our estimate. The obvious question arises, which of these estimates are closest to the true excess mortality? As pointed out above, the method of Simonsen et al. [14] is problematic for two reasons. First, it does not account for temporal correlation between baseline mortality and influenza excess mortality. The resulting estimates of influenza excess mortality may therefore be confounded. Second, their model makes *a priori *assumptions about the parametric shape of the baseline function; these assumptions may or may not be true. They should, in any event, be validated. The Thompson model [11], which superficially resembles a hybrid between the Simonsen model and the model proposed by Schanzer et al. [17] (or the current model), addresses the issue of temporal confounding by controlling for the proportion of influenza test positives. As pointed out in the Background section, the use of that specific variable to control for influenza mortality may not be appropriate. We compared estimates from the TL model with estimates from the current model. The TL model is based on the Thompson model, but influenza-certified mortality is substituted for proportion positives. Although the resulting estimates were about a sixth higher than our estimates, the seasonal pattern was highly consistent with the pattern seen with the current model. This consistency implies relative robustness of excess deaths estimates to the choice of a specific baseline function. The vast difference between our and Thompson's estimates [11] can therefore not be explained by differences in model structure, nor in the way the baseline function is modeled. They may rather be due to the use of proportion of specimens testing positive to control for influenza mortality.

Schanzer et al. [17], like us, used a Poisson model with linear (rather than logarithmic) link function, to analyze weekly mortality data from Canada. Modeling weekly mortality has the advantage of giving higher temporal resolution to the analysis. On the other hand, deaths associated with, but not attributed to influenza may occur with some delay and may thus be partially decoupled from influenza-certified mortality. However, Schanzer and colleagues did not find an obvious lag between weekly influenza-certified mortality and mortality due to other causes. Future studies will be needed to determine what level of temporal aggregation results in the best estimates.

To take into account random variability in influenza-certified mortality, we used a hierarchical model. While the point estimates for *ϕ *(corresponding to *β*_3 _in [17]) obtained from a GLM are very similar to the ones obtained from the hierarchical model (21.35 and 21.16, respectively, for 65+), the confidence limits are much wider for the latter (95% credible interval 18.06, 24.32 vs. Wald 95% confidence interval 20.91, 21.80). This may even be more pronounced for weekly data, where numbers of influenza-certified deaths are often quite small. To the extent that our hierarchical model takes into account random variability of influenza-certified deaths and thus leads to wider confidence limits around the resulting excess mortality estimates, it is more conservative than non-hierarchical GLM models.

**Figure 5:**
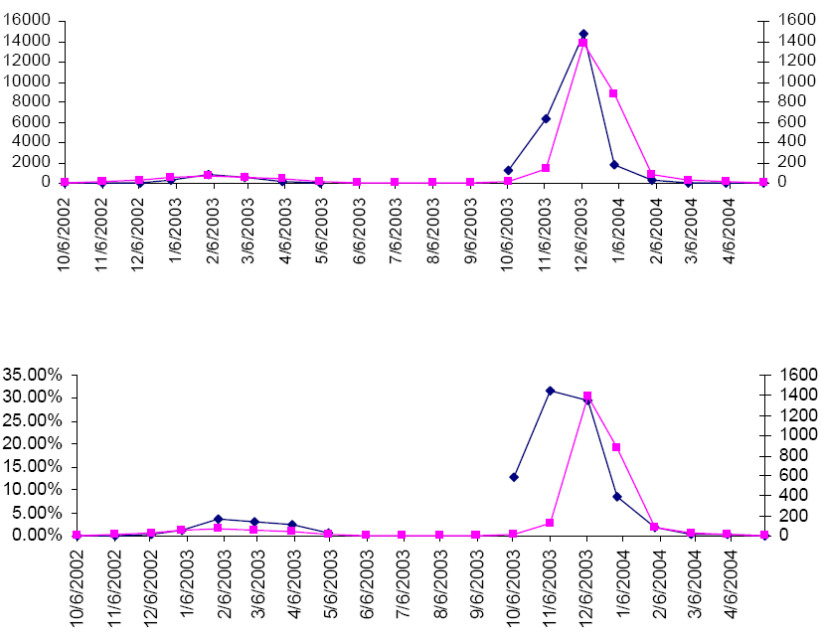
**Number of influenza H3N2 isolates and influenza-certified deaths, U.S., 2002 to 2004**. Upper panel: Number of monthly H3N2 (and proportional unsubtyped) isolates (blue, left y-axis) and influenza-certified deaths (pink, right y-axis) during influenza seasons 2002/03 and 2003/04. Lower panel: Proportion of monthly H3N3 pos. specimens (blue, left y-axis) and influenza-certified deaths (pink, right y-axis).

The potentially most serious shortcoming of our approach to influenza excess mortality estimation relates to the possibility that influenza-certified mortality is a poor indicator of total influenza mortality. Although a death certificate diagnosis of influenza will likely only be given under strong suspicion of that cause, the diagnosis will rarely have been laboratory-confirmed and therefore is likely of low specificity. As this indicator explains mortality in excess of the seasonal baseline very well one could speculate that a better indicator of influenza mortality would lead to even lower estimates of influenza excess mortality. A preliminary comparison of the number of influenza A(H3N2) isolates with influenza-certified death for two influenza seasons (2002/03 and 2003/04) revealed a remarkably close correspondence of the two indicators (Figure 5). Could it be that the number of influenza isolates informs the death certificate diagnosis? The cross-validation of various indicators for influenza mortality will be an important target of future research. The very high proportion of all-cause mortality explained by our model makes it appear quite unlikely that a substantially better model of all-cause mortality can be constructed. We therefore believe that our estimates of excess mortality are better than previous estimates, which are invariably larger than ours. Future studies of excess mortality due to influenza should also consider the possibility that the relationship between the chosen influenza indicator and total influenza excess mortality might change from season to season or even over the season. Studies examining the public health impact of influenza should address some additional issues. First, mortality may not be a good measure of the burden a disease inflicts upon a population. It may be that most deaths that are triggered by an influenza infection occur in people on the verge of dying from other causes, so that the time of death is advanced only by a short period of time. Clearly, the burden of disease would be much lighter in that case than if most people were to die from influenza prematurely by many years. A more adequate measure of disease burden therefore may be disability-adjusted life years (DALYs) [25]. By estimating a more refined age distribution of those who died from influenza, calculations of DALYs, or at least of potential years of life lost (PYLL), should be relatively straightforward. As persons with underlying illnesses are particularly vulnerable to fatal influenza infection, and also might have reduced life expectancy at the time of death, resulting PYLL estimates might be inflated. Second, by modeling monthly mortality independent of mortality during previous periods we ignore, like others, the demographic process that may lead to reduced mortality after epidemic mortality: Not only will lower mortality result from the depletion of the population at risk (smaller denominator), but influenza is also likely to disproportionately affect the frailest individuals, thus leaving the remaining population less frail and thus less susceptible. The first part of this problem could be remedied by modeling the mortality rates rather than numbers of deaths. The second one could be addressed by making specific assumptions on the frailty distribution in the population and on the association between that frailty characteristic and relative mortality risk.

## Conclusion

Previous estimates of excess mortality due to influenza may be biased and inflated. We propose a two-level hierarchical Poisson model where the baseline mortality varies with time. The goodness-of-fit statistic indicates that this model fits the data very well, explaining well above 90% of the observed variation of all-cause mortality during the eleven years study period. The resulting estimates are therefore likely of high validity. Future attempts to quantify the public health burden of influenza should also explore demographic approaches that take into account life expectancy.

## Competing interests

The authors declare that they have no competing interests.

## Authors' contributions

IMF conceived the paper, conducted analyses and wrote the manuscript. MMH provided statistical expertise and contributed to the manuscript.

## Supplementary Material

Additional file 1 **Bayesian analysis of influenza mortality, U.S., 1995–2005 using WinBUGS.** Click here for file
