# Supplementary material for: Revised estimates of influenza-associated excess mortality, United States, 1995 through 2005
Source: Emerg Themes Epidemiol. 2008 Dec 30;5:26. doi: 10.1186/1742-7622-5-26 (PMC2628891; doi:10.1186/1742-7622-5-26)
Supplement: Additional file 1 — Bayesian analysis of influenza mortality, U.S., 1995–2005 using WinBUGS. [file 1742-7622-5-26-S1.zip › readme.doc]

### Explanations to supplemental files for “Revised Estimates of Influenza–Associated Excess Mortality, United States, 1995 through 2005” by Foppa and Hossain, 2008

This appendix contains the code for the MCMC estimation described in this manuscript, as well as the output on which reported results were based.

All MCMC simulation were performed in WinBUGS, which is available free of charge from <http://www.mrc-bsu.cam.ac.uk/bugs/winbugs/contents.shtml>. Calculations were done in batch mode, using R, which is also available at no cost at <http://www.r-project.org/>. To invoke WinBUGS from R, the RBUGs package has to be installed in R. Several other packages may have to be installed as well.

The file “calculations 8-15-2008.txt” contains all data and R code necessary. The code can be cut and pasted into R. However, the file locations of the WinBUGS models need to be specified instead of the “…”.

The results of the MCMC runs on which the result section is based are located in the files “log AG0 8-15-2008.txt” through “log AG3 8-15-2008.txt” and “log AG0 Thompson 8-17-2008.txt” through “log AG3 Thompson 8-17-2008.txt”.

The files “R-squared calculations 8-17-2008.txt” and “R-squared calculations Thomson 8-17-2008.txt” contain the R code for the Bayesian R-squared calculations. The names of the 3 coda files, which contain the data from the three chains, need to be edited.
